# Development of a multiplex loop-mediated isothermal amplification method for the simultaneous detection of *Salmonella* spp. and *Vibrio parahaemolyticus*

**DOI:** 10.1038/srep45601

**Published:** 2017-03-28

**Authors:** Ningwei Liu, Dayang Zou, Derong Dong, Zhan Yang, Da Ao, Wei Liu, Liuyu Huang

**Affiliations:** 1 Institute of Disease Control and Prevention, Academy of Military Medical Sciences, Beijing, 100071, China

## Abstract

Rapid detection of food-borne pathogens is important in the food industry, to monitor and prevent the spread of these pathogens through contaminated food products. We therefore established a multiplex real-time loop-mediated isothermal amplification (LAMP) assay to simultaneously detect and distinguish *Salmonella* spp. and *Vibrio parahaemolyticus* DNA in a single reaction. Two target sequences, one specific for *Salmonella* and the other specific for *Vibrio parahaemolyticus*, were amplified by specific LAMP primers in the same reaction tube. After amplification at 65 °C for 60 min, the amplified products were subjected to melting curve analysis and thus could be distinguished based on the different melting temperatures (*T*_*m*_ values) of the two specifically amplified products. The specificity of the multiplex LAMP assay was evaluated using 19 known bacterial strains, including one *V. parahaemolyticus* and seven *Salmonella* spp. strains. The multiplex LAMP showed 100% inclusivity and exclusivity, and a detection limit similar to that of multiplex PCR. In addition, we observed and corrected preferential amplification induced by what we call LAMP selection in the multiplex LAMP reaction. In conclusion, our assay was rapid, specific, and quantitative, making it a useful tool for the food industry.

Food-borne diseases, caused mainly by food-borne pathogens, have become a major public health issue in both developed and developing countries. It is estimated that 76 million people fall ill and 5,000 die annually from food-borne illness, and a large proportion of these cases can be attributed to *Salmonella* spp. and *Vibrio parahaemolyticus*^1^. In the active etiological surveillance for foodborne diseases in Guangdong, China, during 2013 and 2014, the detection rate of *Salmonella* was highest, followed by that of *V. parahaemolyticus*^2^. *Salmonella* spp. has been identified as the most frequent cause of food-borne infection outbreaks in many countries, and *V. parahaemolyticus* has emerged as a vital food-borne pathogen worldwide due to consumption of raw or undercooked seafood^3^,^4^. Thus, rapid and sensitive detection of *Salmonella* spp. and *V. parahaemolyticus* is required for prevention and timely treatment.

Various methods have been developed to detect *Salmonella* spp. and *V. parahaemolyticus*, including convention culture-based, immunology-based, and molecular methods. Conventional culture-based methods, conducted by selective isolation of bacteria and biochemical identification, are safe but laborious and time-consuming. Immunology-based methods shorten the detection time but are not very effective in terms of detection sensitivity^5^,^6^. Molecular methods such as PCR-based approaches and DNA micro-array, which have been used in the detection of numerous food-borne pathogens, including *Salmonella* and *V. parahaemolyticus*, are labor-saving, sensitive, and specific^7^,^8^, but the need for expensive instruments and trained personnel prevent them from being widely used.

Loop-mediated isothermal amplification (LAMP), invented in 2000 by Notomi *et al*., is a novel nucleic acid amplification method with high sensitivity, specificity, and rapidity for the low-cost detection of pathogens^9^. With 4~6 specifically designed primers that target 6~8 distinct regions of the target gene, a large amount of DNA can be synthesized under a constant temperature in less than 60 min, and the amplified products can be detected by the naked eye. As a result, the LAMP assay has been widely applied in the detection of pathogenic bacteria, viruses, and parasites^10^,^11^,^12^,^13^. However, restriction enzyme analysis of amplified products and probe-based methods, which has been attempted, is laborious and complex^14^,^15^,^16^. Furthermore, the LAMP assay can only detect a gene in a single reaction, and multiplex LAMP assays that can detect and discriminate two or more target genes in a single reaction are limited. In this study, a multiplex LAMP assay was developed to simultaneously detect *Salmonella* spp. and *V. parahaemolyticus* based on different melting temperatures determined by melting curve analysis of amplified products.

## Results

### Optimization of primer concentration in the multiplex LAMP

**Figure 1:**
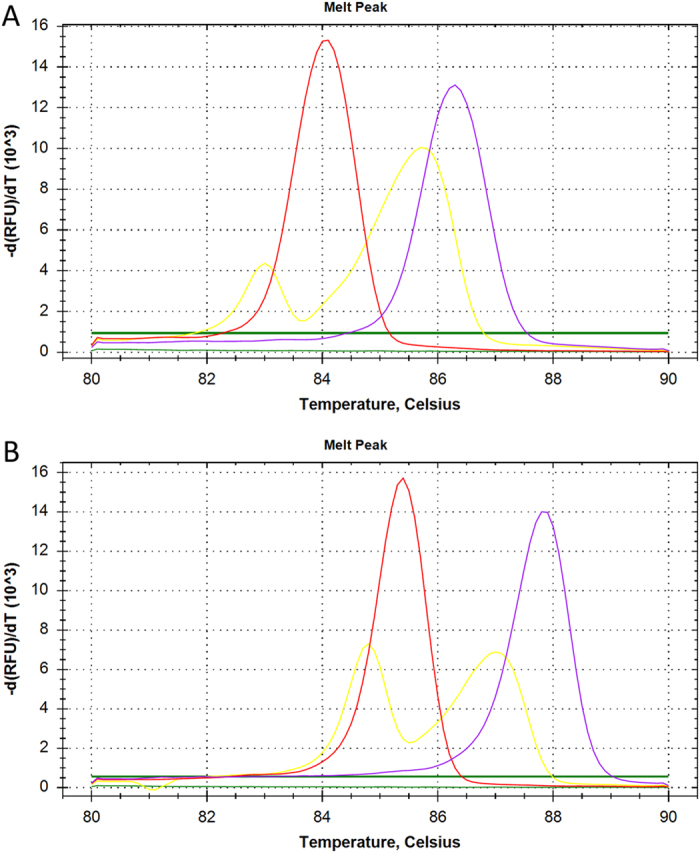
Effect of primer concentration on the discrimination of *Salmonella typhimurium* and *Vibrio parahaemolyticus* target amplification by different *T*_*m*_ values generated by melting curve analysis of the multiplex LAMP assay. (**A**) Equal concentrations of *Salmonella* and *V. parahaemolyticus* primers. (**B**) Halved concentrations of *Salmonella* primers. The melting curve shows temperature on the X-axis and fluorescence on the Y-axis. Well 1 (purple), *S. typhimurium*; well 2 (red), *V. parahaemolyticus*; well 3 (yellow), *S. typhimurium* and *V. parahaemolyticus*; well 4 (green), negative control.

The simultaneous amplification of *Salmonella* and *V. parahaemolyticus* DNA targets was evaluated. Figure 1A shows the results when equal concentrations of *Salmonella* and *V. parahaemolyticus* primers were used. For *S. typhimurium* DNA, the mean *C*_*q*_ and *T*_*m*_ values were 13.96 min and 86.25 ± 0.06 °C, respectively (Fig. 1A, well 1), whereas for *V. parahaemolyticus* DNA, the mean C_*q*_ and *T*_*m*_ values were 12.68 min and 84.05 ± 0.06 °C, respectively (Fig. 1A, well 2). For a mixture of *S. typhimurium* and *V. parahaemolyticus* DNAs, the mean *C*_*q*_ value was 12.14 min and the mean *T*_*m*_ values for *S. typhimurium* and *V. parahaemolyticus* were 85.63 ± 0.15 °C and 82.88 ± 0.21 °C, respectively (Fig. 1A, well 3). No amplification occurred in the negative controls. These results indicate that the multiplex LAMP reaction with two sets of primers for *S. typhimurium* and *V. parahaemolyticus* successfully amplified the two target genes, and the amplified products showed different *T*_*m*_ values (86.25 ± 0.06 °C and 84.05 ± 0.06 °C). In addition, the simultaneous amplification of the target genes from a mixture of *S. typhimurium* and *V. parahaemolyticus* DNAs resulted in two detection peaks, which could be differentiated by their different *T*_*m*_ values (85.63 ± 0.15 °C and 82.88 ± 0.21 °C). However, the fluorescence produced by *V. parahaemolyticus* DNA amplification was clearly weaker than that produced by *S. typhimurium* DNA amplification, suggesting that there was preferential amplification of the *S. typhimurium* target sequence over the *V. parahaemolyticus* target sequence in the multiplex LAMP reaction. Thus, an adjustment of the relative concentration of the primers was required. The concentrations of *S. typhimurium* primers were reduced to half (1.3 μM) and concentrations of *V. parahaemolyticus* primers were kept unchanged, enabling similar amplification efficiency and more intuitive identification of the two target sequences (Fig. 1B). Therefore, these reaction concentrations (1.3 μM for *S. typhimurium* primers and 2.6 μM for *V. parahaemolyticus* primers) were selected for subsequent analysis.

### Specificity of the multiplex LAMP assay

To evaluate the specificity of the multiplex LAMP assay, all 19 bacterial strains were subjected to the multiplex LAMP reaction. The assay successfully detected *Salmonella* spp. and *V. parahaemolyticus*. The *T*_*m*_ value for *V. parahaemolyticus* was 83.8 °C and the *T*_*m*_ values for seven *Salmonella* spp. consistently fell between 86.5 °C and 86.8 °C, with an average of 86.61 ± 0.11 °C. For non-*Salmonella* spp. and non-*V. parahaemolyticus* strains, no *T*_*m*_ value was obtained, suggesting that no amplification occurred. We conclude that the two sets of primers are highly specific for the detection of *Salmonella* spp. and *V. parahaemolyticus* (Table 1).

### Sensitivity of the multiplex LAMP assay

**Figure 2:**
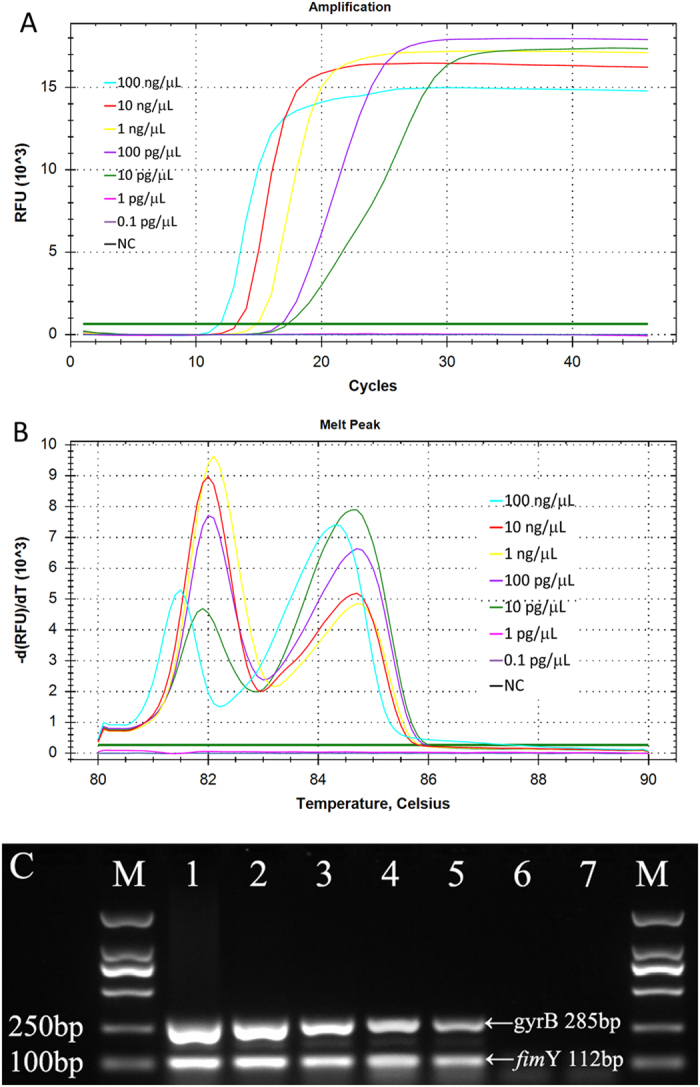
Sensitivity of the multiplex LAMP compared with multiplex PCR for the simultaneous detection of *Salmonella typhimurium* and *Vibrio parahaemolyticus*. 10-fold serial dilutions of *S. typhimurium* (CICC 21483) and *V. parahaemolyticus* (ATCC 17802) DNA templates from 100 ng/μL to 0.1 pg/μL were tested. (**A**) The amplification curve generated from 100 ng/μL to 10 pg/μL shows time on the X-axis and fluorescence on the Y-axis. (**B**) Two melting peaks (84.62 ± 0.18 °C for *S. typhimurium* and 81.90 ± 0.23 °C for *V. parahaemolyticus*) distinguishing *S. typhimurium* and *V. parahaemolyticus* amplification products were generated by melting curve analysis. (**C**) Sensitivity of the multiplex PCR for simultaneous detection of *S. typhimurium* and *V. Parahaemolyticus*. Lanes 1–7: *S. typhimurium* and *V. parahaemolyticus* DNA concentrations of 100 ng/μL, 10 ng/μL, 1 ng/μL, 100 pg/μL, 10 pg/μL, 1 pg/μL and negative control.

To estimate the detection limit of the multiplex LAMP assay, 10-fold serial dilutions of *S. typhimurium* CICC 21483 and *V. parahaemolyticus* ATCC 17802 DNA templates with concentrations ranging from 100 ng/μL to 0.1 pg/μL were used in the amplification reaction. The assay sensitivity for both the simultaneous detection of *S. typhimurium* and *V. parahaemolyticus* and the multiplex PCR was found to be 10 pg/μL (Fig. 2).

### Quantitative capability of the multiplex LAMP assay

**Figure 3:**
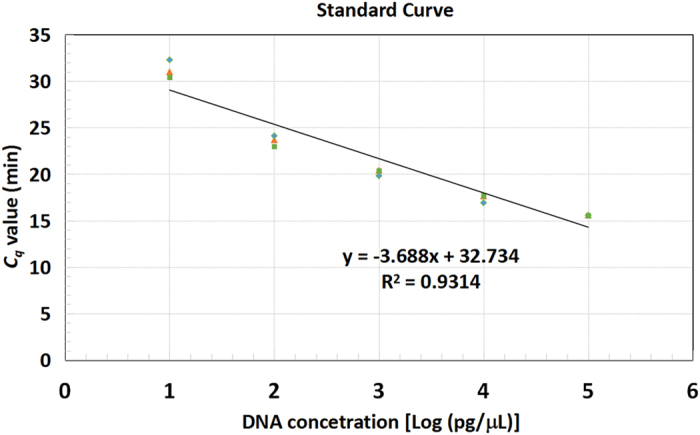
Standard curve generated when simultaneously detecting *Salmonella typhimurium* and *Vibrio parahaemolyticus*. DNA templates with concentrations ranging from 100 ng/μL to 10 pg/μL were tested in triplicate. The standard curve shows DNA concentrations in log scale on the X -axis and *C*_*q*_ values (min) on the Y -axis.

The quantitative capability of the multiplex LAMP assay was also evaluated. Figure 3 shows the standard curve generated when simultaneously detecting *S. typhimurium* and *V. parahaemolyticus* in three independent replicates. In the multiplex LAMP assay, the mean *C*_*q*_ values decreased linearly with increasing target concentrations, and the quantification equation for the assay was validated to be Y = −3.688x + 32.734, with a correlation coefficient (*R*^*2*^) of 0.9314. Moreover, comparison of *C*_*q*_ values for each concentration in all three independent experiments showed minimal variation, indicating that the multiplex LAMP amplification is highly reproducible.

## Discussion

In this report, a simple and rapid protocol for simultaneous detection of *Salmonella* spp. and *V. parahaemolyticus* based on the multiplex real-time LAMP technique was developed. This protocol incorporates the melting curve analysis, which allows the detection and discrimination of amplified products from a mixture by their different *T*_*m*_ values within 60 min. In addition, the phenomenon of amplification bias, defined as uneven or preferential amplification of one target gene over another in a multiplex reaction, which was induced by what we call LAMP selection and can be balanced by primer concentration adjustments, was also observed in the present study.

Considerable time and effort can be saved by simultaneously amplifying and detecting two or more target sequences in a single reaction. However, multiplex LAMP in which multiple sequences can be amplified is limited owing to the difficulty of distinguishing the origin or specificity of the amplified products from the mixture. Several methods have been tried to resolve this difficulty, such as species-specific identification after restriction enzyme digestion^14^,^15^, visual detection by addition of fluorescence-labeled probes or primers coupled with cationic polymers^16^,^17^,^18^, colorimetric distinction using an immunochromatographic strip^19^, and melting curve analysis of amplified products^20^. The last approach was chosen in this study because of its simplicity, rapidity, and specificity. The detection assay takes advantage of the different *T*_*m*_ values of target sequences, which are relatively stable and determined by melting curve analysis. Unlike PCR, the *T*_*m*_ values of LAMP products are closely related to GC content rather than the length of target sequences. As shown in Fig. 1, two target sequences were simultaneously amplified with mean *T*_*m*_ values for *S. typhimurium* and *V. parahaemolyticus* in mixed products of 85.63 ± 0.15 °C and 82.88 ± 0.21 °C, respectively, and were thus clearly distinguished.

The presence of two or more primer pairs in the multiplex LAMP increases the chance of obtaining false positive amplification, primarily because of 4~6 primers contained in a single LAMP reaction, which is another factor limiting the development of multiplex LAMP. Although there is no theoretical limit to the number of primers to be used in a multiplex LAMP reaction, spurious-prone amplification among primers limits the establishment of specific reactions. A multiplex LAMP assay combining 21 primers targeting four *Candida* species has been reported^21^. The high specificity of primers used in this study has been reported and validated previously^10^,^12^, and there was no spurious amplification products in our multiplex LAMP reaction, which showed 100% inclusivity and 100% exclusivity for the detection of 19 bacterial strains including *Salmonella* spp. and *V. parahaemolyticus*. The detection limit for simultaneous detection of *S. typhimurium* and *V. parahaemolyticus* was found to be 10 pg/μL, which was identical to that of LAMP amplification only for *S. typhimurium* or *V. parahaemolyticus*. Additionally, the sensitivity of multiplex LAMP is similar to that of multiplex PCR. The feasibility of the multiplex LAMP was evaluated and validated by the artificially inoculating *Salmonella* and *V. parahaemolyticus* into pasteurizad milk sample. The result showed that the assay could detect and distinguish *Salmonella* and *V. parahaemolyticus* contamination of food samples. Further work to optimize the detection of these two pathogens from clinical samples will also be important.

The quantitative capability of LAMP has been examined previously^12^,^22^. In the present study, a standard curve was generated when detecting *S. typhimurium* and *V. parahaemolyticus* DNAs. In our multiplex LAMP assay, the *C*_*q*_ values decreased linearly with increasing initial concentrations of template DNAs from 10 pg/μL to 100 ng/μL. The standard curve had a linear relationship with *R*^*2*^ value of 0.9314, indicating the quantitative capability of the multiplex LAMP assay.

Ideally, all target sequences in a multiplex detection assay should have similar amplification efficiencies so that greater accuracy of species identification can be obtained. To our knowledge, very few reports have focused on the amplification efficiencies in the multiple amplification system. Amplification bias of one target gene over another was observed in this paper when equimolar primer concentrations were used. The results showed the preferential amplification of the *Salmonella* target sequence over that of *V. parahaemolyticus*, which was induced by what we call LAMP selection^23^. Therefore, adjustment of primer concentration in the multiplex reaction was required, and we found that halving the concentrations of the *Salmonella* primers led to equal amplification efficiency of both target sequences. Generally, optimal concentration of the primers in a multiplex detection reaction may vary greatly between targets and is established empirically^24^.

For the past few years, diseases caused by food-borne pathogens have become a significant public health issue globally^1^. As a leading cause of food-borne disease, *Salmonella* spp. and *V. parahaemolyticus* contamination of food products has become a vital concern for food safety. The multiplex real-time LAMP assay described here can simultaneously detect and distinguish *Salmonella* spp. and *V. parahaemolyticus* based on their different melting temperatures. We also observed preferential amplification in the multiplex LAMP reaction, which was balanced by primer optimization. The assay showed sensitivity similar to that of PCR and was rapid, specific, and quantitative, making it a useful tool for the food industry.

## Materials and Methods

### Bacterial strains, culture conditions, and DNA preparation

In total, 19 known bacterial strains, including a *V. parahaemolyticus* reference strain (ATCC 17802) and seven *Salmonella* sp. strains, were analyzed in this study (Table 1). All strains were stored in 10% (w/v) glycerol broth at −70 °C. *Salmonella typhimurium* CICC 21483 and *V. parahaemolyticus* ATCC 17802 strains were used for primer optimization and sensitivity testing. *Vibrio* strains were cultured in trypticase soy broth supplemented with 2% NaCl at 35 °C overnight. Non *Vibrio* strains were grown in Luria-Bertani broth at 37 °C.

Genomic DNA extraction was conducted using the boiling method. Briefly, 1 ml of overnight bacterial culture was centrifuged at 5,000 × *g* for 10 min and the pellet was suspended in 200 μl of TE buffer [10 mM Tris, 0.1 mM EDTA (pH 8.0)]. The bacterial suspension was boiled for 10 min and centrifuged at 12,000 × *g* for 5 min at 4 °C. The supernatant was used as the DNA template for multiplex LAMP and multiplex PCR assays. The genomic DNA of *S. typhimurium* CICC 21483 and *V. parahaemolyticus* ATCC 17802 was extracted with the Genomic DNA Isolation Kit (Sangon Biotechnology, Shanghai, China) following the manufacturer’s instructions.

### LAMP primers and the multiplex LAMP reaction

We used previously reported *Salmonella*- and *V. parahaemolyticus*-specific primers^10^,^12^ (Table 2). The primers were synthesized by Sangon Biotech (Shanghai). Aliquots (50 μM) of each primer were prepared and used for the multiplex LAMP reaction.

The multiplex LAMP reaction was carried out in a final volume of 25 μL containing 12.5 μL 2 × LAMP reaction buffer [20 mM Tris-HCl, 10 mM KCl, 10 mM (NH_4_)_2_SO_4_, 8 mM MgSO_4_, 0.1% Tween 20, 0.8 M betaine (Sigma-Aldrich), 1.4 mM of each dNTP], 8 U of *Bst* DNA polymerase (New England Biolabs, Ipswich, MA), the *bcfD* and *toxR* primer mix (0.8 μM of FIP and BIP, 0.4 μM of LB and LF, 0.1 μM of F3 and B3), and 2 μL of DNA template. Additionally, 1 μL EvaGreen (20× in water) (Yeasen Biotech, Shanghai, China) was added. The reaction was performed at 65 °C for 40 min, followed by melting curve analysis from 80 °C to 90 °C with 0.1 °C increment per second. Each experiment was performed in triplicate.

### Primer optimization

Initially, equimolar primer concentrations of 2.6 μM each were tested in the multiplex LAMP reaction. In addition, equimolar template concentrations at 100 ng/μL of DNA isolated from *S. typhimurium* CICC 21483 and *V. parahaemolyticus* ATCC 17802 were provided to the reaction to keep the same primer-to-template ratio. The amplification was performed in quadruplicate and the results are shown as *C*_*q*_ values, which represent the cycle in which fluorescence is first detected, and *T*_*m*_ (melting temperature) values determined by the melting curve analysis. A no-template control, in which double-distilled water was substituted for template DNA, was used in each amplification.

### Multiplex PCR amplification

Multiplex PCR was performed with the specific primers for simultaneous detection of *Salmonella* spp. and *V. parahaemolyticus*. PCR primers for *Salmonella* were *fim*Y-F (CCGTATGGCTGGGCGTTT) and *fim*Y-R (AGTACGGCTAAAGCTTTCCGATAAG), and PCR primers for *V. Parahaemolyticus* were *gyr*B-F (CGGCGTGGGTGTTTCGGTAGT) and *gyr*B-R (TCCGCTTCGCGCTCATCAATA). The reaction was carried out in a 25 μL volume with 12.5 μL Taq Mix (TaKaRa, Dalian, China), 1 μL each of forward and reverse primers (10 μM), and 2 μL of DNA templates at 100 ng/μL to 1 pg/μL. The multiplex PCR amplification was conducted as follows: initial denaturation at 95 °C for 5 min, 35 cycles of 95 °C for 30 s, 60 °C for 30 s, 72 °C for 30 s, and a final extension at 72 °C for 5 min. The amplified products were analyzed by electrophoresis on 1.5% agarose gels.

## Additional Information

**How to cite this article:** Liu, N. *et al*. Development of a multiplex loop-mediated isothermal amplification method for the simultaneous detection of *Salmonella* spp. and *Vibrio parahaemolyticus.* *Sci. Rep.* **7**, 45601; doi: 10.1038/srep45601 (2017).

**Publisher's note:** Springer Nature remains neutral with regard to jurisdictional claims in published maps and institutional affiliations.

## Figures and Tables

**Table 1:** Bacterial strains used in this study along with the specificity of the multiplex LAMP assay for the detection of *Salmonella* spp. and *Vibrio parahaemolyticus*.

No.	Bacterial species	Strain serial	Multiplex LAMP
1	*Vibrio parahaemolyticus*	ATCC17802	+
2	*Salmonella typhimurium*	CICC21483	+
3	*Salmonella typhimurium*	ATCC14208	+
4	*Salmonella enteritidis*	1655	+
5	*Salmonella enteritidis*	CMCC(B)50335	+
6	*Salmonella choleraesuis*	SH1055	+
7	*Salmonella aberdeen*	SF080	+
8	*Salmonella senftenberg*	2638	+
9	*Listeria monocytogenes*	ATCC19115	−
10	*Shigella flexneri*	4536	−
11	*Shigella sonnei*	2531	−
12	*Shigella dysenteriae*	CMCC(B)51105	−
13	*Staphylococcus aureus*	CMCC(B)26003	−
14	*Escherichia coli*	ATCC25992	−
15	*Escherichia coli O157*	NCTC12900	−
16	*Stenotrophomonas maltophilia*	K279a	−
17	*Proteus vulgaris*	CMCC49027	−
18	*Vibrio cholera*	3802	−
19	*Yersinia enterocolitica*	027	−

**Table 2:** Primer sequences for the multiplex LAMP assay.

Primer name	Primer Sequence (5′–3′)	Reference
*bcfD*-F3	CCGGACAAACGATTCTGGTA	10
*bcfD*-B3	CCGACATCGGCATTATCCG	
*bcfD*-FIP	TGCACTTTACCGGTACGCTGAA-TACAGCGGCAATTTCAACCA	
*bcfD*-BIP	CGGTCTGGATTCGCAGGTCAAA-GCGATAGCCTGGGGAAC	
*bcfD*-LF	TACCCCCTCCGGCTTTTG	
*bcfD*-LB	ACAATGCGTCTTATCGCTACG	
*toxR*-F3	CGAAGTTGTACGATTAGGAAG	12
*toxR*-B3	AAACTCTGGAGATTTGGTTG	
*toxR*-FIP	GCTCGTTACGGGTTAAAACTTCG-CAACGAAAGCCGTATACTCC	
*toxR*-BIP	GGCGTGAGCAAGGTTTTGAG-CCTTCAACATCTTACGCAG	
*toxR*-LF	GGTCTCTCCGCCAACATCA	
*toxR*-LB	GTGGATGACTCAAGCCTGACT	
